# Notes on Preparations for the Sick

**Published:** 1901-03

**Authors:** 


### Notes on Preparations for the Sick.

Several new *preparations of iron*, in various combinations, in the form of glycerophosphates, by H. E. Matthews, Clifton, have been submitted to us:—

**Syrupus Ferri Glycerophosphatis** (Matthews).—Dose, ½ to 1 fluid drachm. A pleasantly-flavoured syrup, containing 2 grains of iron-glycerophosphate in each fluid drachm.

**Syrupus Ferri et Quininæ Glycerophosphatis** (Matthews).—Dose, ½ to 1 fluid drachm. Each fluid drachm contains 2 grs. iron-glycerophosphate and $\frac{1}{12}$ grain quinine.

**Syrupus Glycerophosphatum Co.** (Matthews).—Improved chemical food. Contains the glycerophosphates of iron, calcium, sodium, and potassium, in the same proportions as the phosphates in Parrish's Chemical Food. The preparation is agreeable to the palate, without pronounced styptic taste, and is almost neutral in reaction. The strong acidity of phosphate syrups is often an objection to their administration. These glycerophosphate preparations have the conspicuous advantage of practical neutrality and of presenting the bases in a directly assimilable form. They can be administered with a minimum of risk to the teeth.

**Egg Emulsion of Cod Liver Oil with Glycerophosphates** contains 50 per cent. cod liver oil, emulsified with 10 per cent. of yolk of egg, and containing in each fluid ounce calcium glycerophosphate and sodium glycerophosphate, of each 8 grains. Dose, 2 to 8 fl. drachms.

The difficulty of prescribing fluid preparations of cinchona, owing to the unpleasant appearance of a sediment, is obviated by the use of **Liquor Cinchonæ Detannatus** (MATTHEWS) prepared from the bark of *Cinchona ledgeriana* (Java calisaya), and standardised to contain 5 per cent. of the mixed alkaloids in combination with hydrobromic acid. A series of analyses shows that the average proportion of quinine to other alkaloids is higher in *cinchona ledgeriana* than in any other of the quinine-yielding barks.

The preparation is freed from cincho-tannic acid and cinchona red, and gives a perfectly clear solution when mixed with water. It is stable in neutral and acid solution; alkalis in excess precipitate the alkaloids.

It is useful in all cases where the action of cinchona alkaloids is desired and the astringent bodies of the bark are objectionable. The combination of the alkaloids with hydrobromic acid tends to prevent cinchonism.

---

Mr. C. H. Whiteford (Plymouth) has sent us a **substitute for a flat sponge** made of camel-hair blanketing, covered with butter-cloth. It is easily sterilised by boiling, has great power of absorption, and is soft and elastic. The cost of the material for each "sponge" is about 2½d.

---

**"Johannis" Natural Mineral Water; "Johannis" Potash Water** (grs. vijss. per bottle); **"Johannis-Lithia"** (gr. i. per bottle).—THE APOLLINARIS COMPANY, LIMITED, London.—To the natural water are added definite quantities of lithium bicarbonate and potassium bicarbonate respectively. These waters are pleasant to the palate, and are a convenient vehicle for small doses of drugs which should be of great service to gouty patients. They are intended for regular and continuous dietetic use, and hence the dose is necessarily a small one.

---

**Bovinine.**—THE BOVININE COMPANY, London.—We have received a volume of 256 pages, containing over 400 clinical reports compiled from private and hospital practice respecting the use of Bovinine in medicine and surgery. Special attention is directed to the combined use of Bovinine with either peroxide of hydrogen or hydrozone for the perfect and complete cleansing of skin surfaces to be operated upon, and various combinations with Bovinine have given excellent results in the surgical treatment and dressing of various lesions. Combinations with thymol, with iodoform, and with Thiersch dressing are recommended. Bovinine is no new remedy; it has withstood the experience of many years, and it maintains its well-grounded reputation.

---

**Tabloids.**—Burroughs, Wellcome, & Co., London.—**Beta-Naphthol**, grs. iij.; **Benzo-Naphthol**, grs. v.—The pungent taste and slight solubility of these drugs render the tabloid form especially appropriate. They are both serviceable in cases of gastric fermentation, particularly when there is dilatation of the stomach; also as intestinal antiseptics in enteric fever and dysentery. Antiseptic intestinal treatment has not yet made much progress, but with the help of convenient doses of these drugs in tabloid form it should be possible to diffuse these antiseptic drugs pretty freely along the whole length of the alimentary canal.

**Calomel**, grs. iij.—Tabloids of this drug are prepared in various doses ranging from gr. $\frac{1}{10}$ up to five grains. The three-grain tabloid is a convenient dose when it is desired to obtain the diuretic action of the drug, the dose being given three times a day for three successive days.

**Quinine Bisulphate and Potassium Citrate (Effervescent).**—This tabloid combines in a convenient and agreeable form the antimalarial and antipyretic properties of quinine with a saline febrifuge. Each tabloid contains one grain of quinine bisulphate and fifteen grains of potassium citrate. If powdered before being moistened, the addition of water will produce a refreshing effervescing draught.

**Three Syrups.**—Each tabloid contains the equivalent of fifteen minims of Easton's syrup with fifteen minims of the compound syrup of hypophosphites and thirty minims of compound syrup of phosphate of iron. The instability of the syrups in warm climates renders the tabloid form useful, especially for tropical use, and as the tabloids are sugar coated they form a convenient, portable, and agreeable dose for all patients who are continuing treatment when following the ordinary round of social, professional, or commercial life.

---

**Sanochinol: a Derivative of Quinine.**—A. Koch, Hamburg; Arthur & Co., London.—This non-toxic derivative of quinine seems likely to be of value in the treatment of malaria in tropical countries. It is produced by the action of nascent oxygen on quinine. The product is a yellowish-brown, amorphous, acid salt, readily soluble in water, and tasteless.

